# A Rare Case of Urinary Tract Fungal Ball Leading to Fungemia and Bilateral Chorioretinitis

**DOI:** 10.1155/2020/8828289

**Published:** 2020-09-28

**Authors:** Christopher Ferari, Chad Crigger, Chad Morley

**Affiliations:** Department of Urology, West Virginia University School of Medicine, Morgantown, WV 26505, USA

## Abstract

**Background:**

Fungemia due to obstructive urinary tract fungal ball is exceedingly rare. These patients often have multiple predisposing conditions, including diabetes or antimicrobial exposure. While candiduria can be relatively common in this population, urinary tract fungal balls are a rare entity. Hospitalists should be aware of this rare complication in patients presenting with funguria. *Case Presentation*. We present a case of a 44-year-old male with type II diabetes, chronic hepatitis C secondary to injection drug use, and chronic kidney disease who developed a urinary tract fungal ball leading to fungemia and subsequent bilateral chorioretinitis, additionally complicated by emphysematous cystitis and pyelonephritis. Additional invasive treatment options beyond typical antifungals are often required in the case of urinary tract fungal ball, and in this case, bilateral nephrostomy tubes and micafungin were employed. Hospital course was complicated by *C. tropicalis* fungemia with subsequent bilateral fungal chorioretinitis on dilated fundus exam. This was effectively treated with cyclogyl and prednisolone drops along with bilateral voriconazole injections. Follow-up imaging and cultures showed resolution of fungemia, urinary tract masses, and chorioretinal infiltrates; however, recurrent polymicrobial UTIs continue to be an issue for this patient.

**Conclusions:**

Special multidisciplinary management is required in the treatment of urinary tract fungal balls with subsequent fungemia, including nephrostomy tubes, antifungal irrigation, ureterorenoscopy, and more powerful antifungals such as amphotericin B and 5-flucytosine. This management draws from a myriad of specialties, including urology, infectious disease, and interventional radiology. Additionally, the literature has demonstrated that only approximately half of patients with fungemia receive an ophthalmologic evaluation. Ophthalmologic and urologic cooperation is essential in the case of obstructive uropathy leading to fungemia as the obstructive uropathy must be relieved and these patients should receive a dilated fundus exam.

## 1. Introduction

Funguria is a common finding in hospitalized patients, most notably in the intensive care unit where patients often have multiple predisposing factors such as diabetes mellitus, indwelling catheters, and frequent antimicrobial exposure [1]. Studies have demonstrated a significant prevalence of candiduria in this population ranging from 1.6% to over 20% [2–4]. A urinary tract fungal ball, however, is rare in these patients. We report a case of emphysematous cystitis and pyelonephritis secondary to funguria originating from coexisting bladder and ureteral fungal balls leading to bilateral fungal chorioretinitis.

## 2. Case Presentation

A 44-year-old male with type II diabetes, chronic hepatitis C secondary to injection drug use, and chronic kidney disease presented to our facility as a transfer for suspected emphysematous pyelonephritis. This was initially demonstrated at an outside facility one month prior, at which time a left ureteral stent was placed. Symptoms at presentation included severe left flank and abdominal pain with pneumaturia, hematuria, fevers, and diarrhea. Immediate workup revealed a glucose of 647 mg/dL, hemoglobin A1c of 11.1%, and urine leukocytosis. Antibiotic therapy initially consisted of broad-spectrum meropenem 1 g and linezolid 600 mg, which was switched to fluconazole 200 mg and ertapenem 1 g per infectious disease (ID) recommendations after urine culture grew pan-sensitive *Candida tropicalis*. No bacteria were detected on urine culture.

**Figure 1:**
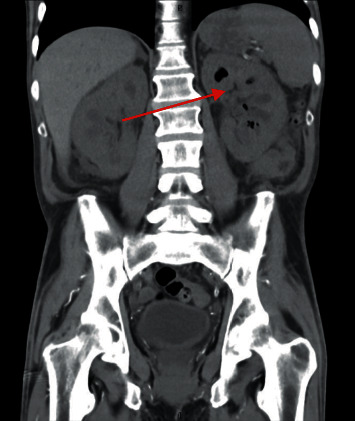
Coronal CT image showing evidence of emphysematous pyelonephritis (red arrow).

**Figure 2:**
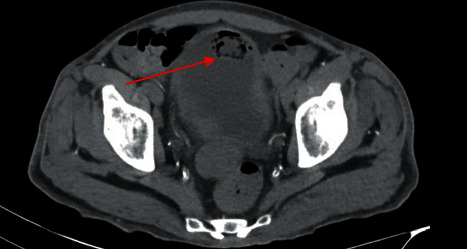
Transverse abdominal CT image showing evidence of bladder fungal ball with emphysematous cystitis (red arrow).

**Figure 3:**
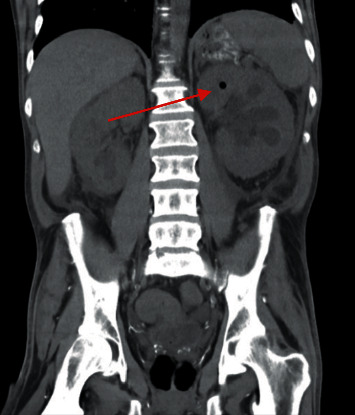
Coronal CT image showing evidence of diminished emphysematous pyelonephritis (red arrow) compared to Figure 1.

**Figure 4:**
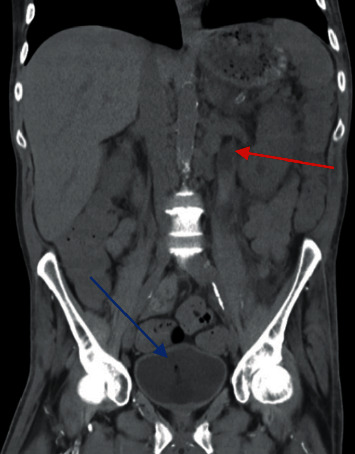
Coronal CT image showing evidence of ureteral fungal ball (red arrow) and emphysematous cystitis (blue arrow).

Cross-sectional imaging revealed bilateral hydronephrosis with significant air in the left renal collecting system as well as concern for emphysematous cystitis with a lesion in the bladder (Figures 1 and 2). He was transferred to our facility 3 days later after repeat imaging showed decreased air in the left renal collecting system and the presence of a new hyperdensity in the left proximal ureter concerning for a fungal ball (Figures 3 and 4). Urine culture once again grew *C. tropicalis*, and antimicrobial therapy was changed to IV micafungin 100 mg. Again, no bacteria were detected on urine culture. Physical exam was noteworthy for bilateral flank pain and significant penile pain. He endorsed methamphetamine and marijuana use, and urine drug screen was positive for opiates and oxycodone.

**Figure 5:**
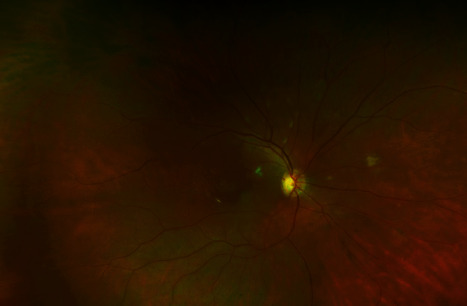
Right fundus showing exudates suspicious for fungal chrioretinitis.

**Figure 6:**
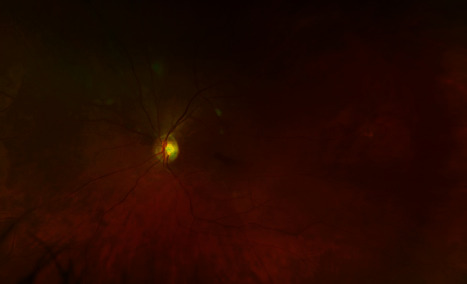
Left fundus showing exudates suspicious for fungal chrioretinitis.

Per urology recommendations, bilateral nephrostomy tubes were placed on the day of transfer and ID deescalated antifungals to fluconazole 200 mg. However, the patient's blood cultures grew *C. tropicalis* on day 3, which prevented his discharge at this time and prompted an escalation of antifungal therapy back to IV micafungin 150 mg. Ophthalmology was consulted per ID recommendations, and dilated fundus exam showed exudates suspicious for fungal chorioretinitis (Figures 5 and 6). Vision symptoms at this time included worsening of ongoing blurry vision as well as occasional black spots in his field of vision. Cyclogyl 1% and prednisolone 1% drops were started, with bilateral voriconazole 100 mcg intravitreal injections performed on day 6. The patient was discharged on day 11 in stable condition with bilateral nephrostomy tubes in place and a six-week course of PO voriconazole 200 mg bid.

Follow-up CT imaging one month after discharge showed resolution of emphysematous cystitis and pyelonephritis. Ophthalmology follow-up showed fading chorioretinal infiltrates. His recovery was complicated by an episode of *C. difficile* colitis three weeks after initial discharge, adequately treated with vancomycin. Since this discharge, the patient has had recurrent polymicrobial UTI issues, growing *Pseudomonas* and methicillin-resistant *S. aureus* (MRSA) on several occasions, some requiring readmission. At a future admission three months after discharge for nephrostomy exchange, nephrostomy cultures grew *Pseudomonas*, which was adequately treated. No further nephrostomy complications were encountered.

## 3. Discussion

Reports of a urinary tract fungal ball leading to separate findings of fungemia, emphysematous pyelonephritis and cystitis, and bilateral fungal chorioretinitis are rare in the literature [5–8]. This patient's urine cultures were negative for bacteria and positive for only *C. tropicalis*, which has been shown to be a gas producer in the literature [9]. We know that diabetes mellitus is a risk factor for emphysematous urinary tract infections [10, 11] and thus could have contributed to this patient's infection. In a retrospective review of patients with candidemia, only 51% of patients were evaluated by an ophthalmologist, of which evidence of ocular candidiasis was present in 12.5% [12]. It is clear that involvement of ophthalmology in cases of fungemia due to urinary tract fungal ball is critical, even with no ocular symptoms present. In lieu of typical first-line antifungals such as fluconazole, urinary tract fungal balls require special multidisciplinary management like nephrostomy tubes, antifungal irrigation, ureterorenoscopy, and more powerful antifungals such as amphotericin B and 5-flucytosine [5, 13–15]. Although chronic echinocandins are not thought of as good drugs for the urinary tract, there are case series and case reports of successful treatment of Candida species with micafungin while subsequently avoiding the toxicity of the previously mentioned antifungals such as amphotericin B [16–18]. Radiologic diagnosis of fungal balls is difficult as they can be mimicked by more common red herrings such as blood clots or inflammatory debris; however, an intraluminal defect coupled with air can be a clue [19].

One of our strengths in the treatment of this case included a relatively early ophthalmology consult in order to prevent future vision complications. An example of a limitation in our approach was the possible deescalation of antifungals too early, as blood cultures were subsequently positive. Hospitalists should be aware of the rare complications of funguria presented here and should not hesitate to involve urology, ophthalmology, and infectious disease specialists when concerning symptoms arise.

## Abbreviations

ID: Infectious disease
UTI: Urinary tract infection
MRSA: Methicillin-resistant *S. aureus.*
